# Migration of digital functional capacity assessments from device resident to cloud-based delivery: Development and convergent validity

**DOI:** 10.1016/j.scog.2024.100331

**Published:** 2024-09-24

**Authors:** Philip D. Harvey, Richard S.E. Keefe, Peter Kallestrup, Sara J. Czaja, Hans Klein, William Horan

**Affiliations:** a University of Miami Miller School of Medicine, Miami, FL, United States of America; b I-Function, Miami, FL, United States of America; c Duke University, Durham, NC, United States of America; d Weil Cornell Medicine, New York, NY, United States of America; e WCG Endpoint Solutions, Cary, NC, United States of America; f Karuna Therapeutics, Boston, MA, United States of America

**Keywords:** Functional capacity, Digital phenotyping, Cognition, Schizophrenia

## Abstract

Decentralized clinical trials are leading to rapid changes in assessment technology, including an expansion of interest in remote delivery. As technology changes, some of the updates include migration to fully cloud-based software and data management, with attendant differences in hardware, response modalities, and modifications in the level of tester engagement. It is rare to see systematic descriptions of the process of migration and upgrading of technology-related assessments. We present comparative data on successive generations of two widely used functional capacity measures, the Virtual Reality Functional Capacity Assessment Tool (VRFCAT) and the Functional Capacity Assessment and Training System (FUNSAT). Four samples of healthy older individuals completed either the original device-resident, computer-administered versions, or cloud-based, tablet-delivered versions of these tasks. For the VRFCAT, performance and correlations with age were similar across versions, although performance was slightly (5 %) faster with iPad delivery. For the FUNSAT, performance and correlations with age and cognitive task scores were generally similar across versions for English Speakers, though there were some differences related to the testing language for the cloud-based version. These results support the feasibility of migrating digital assessments to cloud-based delivery and substantiate fundamental similarity across delivery strategies.

## 1 Introduction

Performance-based assessment of everyday functional skills, commonly referred to as functional capacity (Mantovani and Teixeira, 2015), is an important assessment domain in aging and mental health. These strategies offer the benefit of testing everyday functional skills in a more controlled setting, so that abilities can be assessed independently of factors such as the opportunity or motivation to spontaneously perform the task in daily life. Interventions aimed at improving cognition across several neuropsychiatric conditions are required by regulators to demonstrate their functional relevance (Buchanan et al., 2011), with performance-based assessments proving superior to self and informant reports of abilities in both aging- related conditions (Goldberg et al., 2010; Harvey et al., 2017) and serious mental illness (Durand et al., 2015; Sabbag et al., 2011).

Assessment of functional capacity has evolved from paper and pencil simulations of everyday tasks (Mausbach et al., 2007; Velligan et al., 2007) to current veridical representations of task demands using digital presentation . Digital functional capacity assessments have had several formats, including sequential “story-board” simulations of everyday tasks and modular assessments of individual technology-based functional tasks. The Virtual-Reality Functional Capacity Assessment Tool (VRFCAT™; Keefe et al., 2016) is an example of the first strategy, examining the ability to perform a structured sequence of tasks related to preparing a meal, including developing of a shopping list, taking transportation, shopping in a virtual supermarket, and paying with cash. The Functional Capacity Assessment and Training System (FUNSAT™; Czaja, Loewenstein, Lee, Fu and Harvey, 2017a, Czaja et al., 2017b), an example of the second strategy, assessing 6 different technology-related functional skills, including ATM and internet Banking, Ticket Kiosk Purchase, refilling prescriptions with a telephone voice menu and a pharmacy website, on-line shopping, and medication management. The VRFCAT and FUNSAT share several features, including alternate forms designed for repeated measurement, uniform administration of the instructions delivered by the application to maintain fidelity to administration strategy, and automated scoring of performance (time and error scores).

The VRFCAT and FUNCAT both also have considerable evidence regarding their utility, acceptability, and psychometric properties in healthy, aging, and SMI populations (Czaja, Loewenstein, Lee, Fu and Harvey, 2017a, Czaja et al., 2017b; Keefe et al., 2016; Atkins et al., 2015). Further, both have shown strong evidence of convergence with critical cognitive outcomes and utility as a repeated measure in these two populations (Harvey et al., 2020, Harvey et al., 2021). Finally, and most recently, both have shown evidence of sensitivity to treatment, with the studies showing treatment gains from computerized training interventions targeting cognition and functional skills (Czaja, Loewenstein, Lee, Fu and Harvey, 2017a, Czaja et al., 2017b; Harvey et al., 2024) and social cognition (Nahum et al., 2021). Thus, both systems have shown evidence of all the properties required of a suitable functional capacity outcome measure for use in clinical trials and practice.

The VRFCAT and FUNCAT were initially developed and delivered as device-resident software programs, administered in-person on computers, with tester-supervised administration. As the frequency of remotely delivered interventions increases, including both decentralized pharmacological treatment studies and remote computerized training interventions, targeting cognition, social cognition, and functional skills, modifications in the delivery of outcomes assessments are required. One of the innovations for both VRFCAT and FUNSAT was the migration of the software from device-resident to cloud-based delivery. This innovation markedly reduces administration and operational challenges, as the new generation cloud-based software delivers all instructions, scores performance and automatically uploads scores, and provides maximum administration fidelity. Thus, data entry is automated and instantaneous, requiring no aggregation or management. However, if the migration of the systems was to be associated with substantial differences in performance, then adjustments would be required. For instance, when the paper and pencil Brief Assessment of Cognition in Schizophrenia (BACS; Keefe et al.,2004) was directly migrated to cloud-based iPad® administration, creating the (BAC-App™; Atkins et al., 2017), some re-norming was required because of performance differences with certain tasks across paper vs. digital administration. The chances of this are smaller with a purely digital migration, but empirical data provide the best evidence of convergent validity across delivery methods.

The current paper presents the results of analyses of key outcome variables to examine the comparative results of pre- and post-migration assessments for both VRFCAT and FUNSAT in samples of healthy older adults. Data regarding discrimination of clinical populations from HC have been previously published for both (Atkins et al., 2015; Keefe et al., 2016; Czaja, Loewenstein, Lee, Fu and Harvey, 2017a, Czaja et al., 2017b). The VRFCAT migration included transition to cloud-based delivery of stimuli and change in the delivery platform from a Windows computer to an i-Pad professional tablet. This resulted in a transition from mouse-driven response entry to touchscreen. The device-resident sample presented here included a nationally representative normative sample assessed in person (Atkins et al., 2018) and the migration sample was a group of HC participants in a previously unpublished study of wearable devices in aging, also assessed in person. The VRFCAT has 12 performance objectives, with the critical dependent variable being total time to completion of all objectives, which typically takes about 20–30 min in HCs. Two other variables, total errors, and number of forced progressions (i.e., being automatically progressed to the next objective if an objective is not successfully completed within timely manner), are collected and are relatively uncommon in HC. Convergence with participant age was also evaluated.

The FUNSAT migration also included transitions to fully cloud-based delivery and from mouse to touch-screen response entry, adding as well as fully remotely deliverable assessment and training strategy. There are no device requirements other than screen size (>10 in.) and accessing a commonly used internet browser. Participants in the FUNSAT™ device-resident and migration samples were HC subjects in two separate CCT and functional skills training intervention studies (Czaja, Kallestrup and Harvey, 2020, Czaja, Kallestrup and Harvey, 2024) and were tested at baseline prior to initiation of training. The FUNSAT™ has 6 different tasks, each with 3 to 6 subtasks. Thus, with modular subtasks, there are variations in completion time across individual subtasks, as well as in the total score. We therefore compared performance in terms of total completion time and time to complete individual tasks across delivery systems. Convergence data was available for age and cognitive test performance, with the same cognitive test battery performed at baseline in each study.

## 2 Methods

### 2.1 Participants

Cognitively normal control participants were included in both studies, which were based in Durham, North Carolina (VRFCAT) and FUNSAT sites in Miami for both versions and additional sites in New York City for the cloud-based version. Both projects recruited their participants with similar strategies, through advertisements and an aging research center at Duke University, and at community centers in New York and Miami, targeting participants over the age of 60. For the VRFCAT, a nationally representative sample of older HC had been collected for a normative study that used the device-resident version of the VRFCAT software. For the cloud-based version, HC participants were comparison subjects in a wearable device study. For the FUNSAT studies, cognitively unimpaired individuals were controls in two randomized trials of computerized cognitive and functional skills training, the first of which was an in-person training study with device resident software and the second using the cloud-based version of the assessment software with fully self-administered home-based training and retesting with two alternative forms of the FUNSAT after in person baseline assessments. There was a wider age range sampled in the VRFCAT studies, so we included all participants aged 60 to 90 to match the FUNSAT entry criteria.

All baseline screening and diagnostic assessments were performed prior to administration of the functional capacity simulations. Participants in both locations were screened in person and common exclusion criteria were applied, including cognitive impairments indexed with the Montreal Cognitive Assessment (MOCA; Nasreddine et al., 2005) with cutoff scores at <23 for VRFCAT and < 26/25 for FUNSAT), psychiatric disorders other than a history of major depression, a medical history of brain diseases like CVA, seizures, tumors, or severe traumatic brain injuries with prolonged loss of consciousness, as well as sensory or motor limitations, and inability to read at the 6th grade level in the English (Or Spanish for the cloud-based study for FUNSAT). Local or commercial IRBs approved the study procedures, and all participants signed an informed consent form. No participants who required proxy consent were enrolled and no participants were included in both initial and migration samples.

### 2.2 Functional capacity assessment tasks

#### 2.2.1 Virtual reality functional capacity assessment tool: VRFCAT

**Fig. 1:**
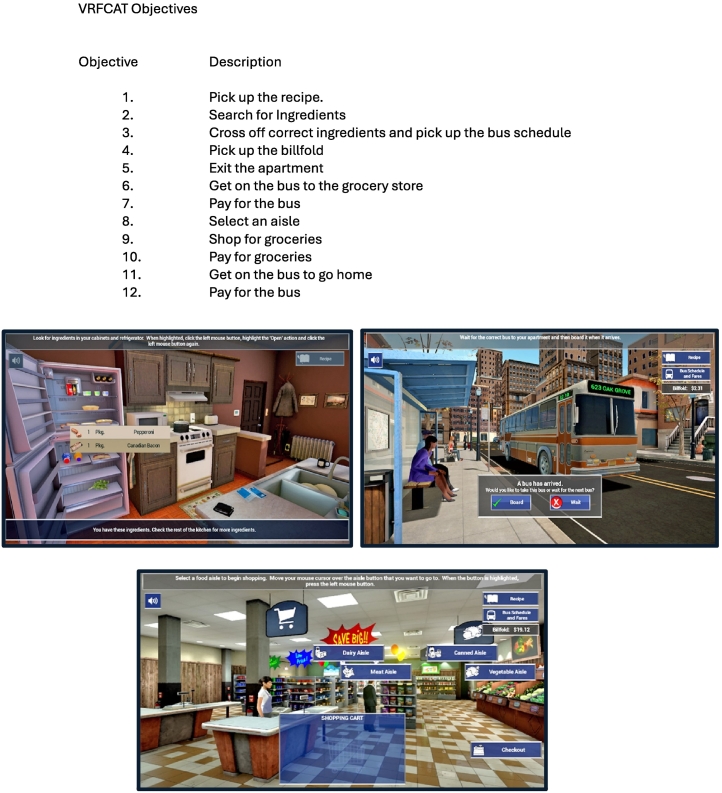
Virtual reality Functional Capacity Assessment Tool (VRFCAT): Objectives and Stimulus Samples.

The VRFCAT (Fig. 1) presents participants with multiple instrumental activities of daily living including: navigating a kitchen, catching a bus to a grocery store, finding/purchasing food in the grocery store, and returning home on a bus. The VRFCAT has 12 performance objectives and participants complete all objectives in order. If a given objective cannot be completed (defined as five errors or more than five minutes spent on a given objective), the participant is forced to progress; and the program then continues along to the next objective. Primary endpoint is Total Adjusted Time (time to complete all objectives, adjusted for instructions and error messages). Prior to completing the VRFCAT, participants complete a brief structured digitally delivered tutorial to familiarize them with the instructions, stimuli, and entering responses. Once the assessment starts, it is self-paced until the end, although participants could request a pause of the assessment. There are six alternative forms of the VRFCAT, with all 6 administered first with equal frequency in the device resident study, evenly distributed across cases, and only form 1 in the tablet-based study.

#### 2.2.2 Functional skills assessment and training system: FUNSAT

**Fig. 2:**
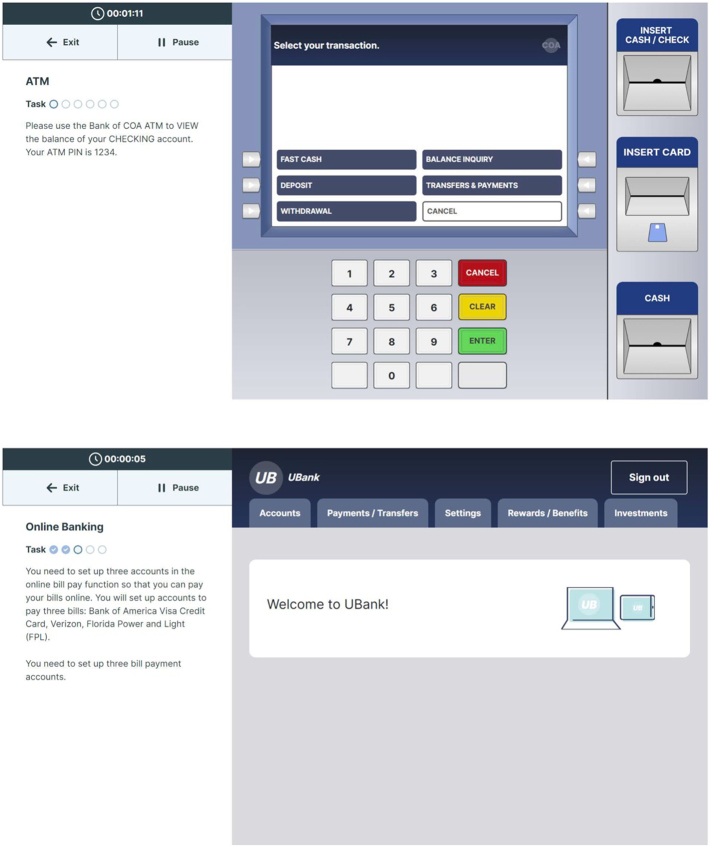
Functional Skills Assessment and Treatment System: Sample Simulations.

The FUNSAT program (Fig. 2) assessed the same skills across both versions of the software, including managing medication (understanding prescription labels and organizing medication with a pill container), shopping at an online pharmacy, using a telephone voice menu for prescription refills, operating an ATM and a ticket kiosk, and internet banking. The six tasks comprised three to six subtasks with sequential requirements, and they were presented in a multi-media style with graphic representations, text, and audio. In the telephone refill task, for instance, participants contacted the pharmacy using a simulated mobile phone keypad (identical in size and appearance to a contemporary smartphone), listened to auditory instructions, selected a preferred delivery method, selected a certain time and day for pick-up, and refilled several prescriptions (pill bottles showed on the screen. Real-time data was gathered on errors and completion times. Only the time the participant was actively working on the activities was included in the completion time, like the VRFCAT. Error feedback appeared as a pop-up window with the original instructions repeated. If responses to an item in a subtask (such as selecting the wrong account in the ATM task) were incorrect four consecutive times, the FUNSAT program automatically progressed to the next question. Forced progressions were not recorded as a separate variable, because they were always triggered by 4 consecutive incorrect responses to an item. The device resident version of FUNSAT had two alternative forms, which were compared in a separate non-interventional study of older NC adults (Kim et al., 2024). The cloud based version has 3 alternative forms, which were administered in a fixed order in the treatment study, with forms B and C being administered on a completely remote basis after the completion of training in the treatment study.

### 2.3 Migration process

#### 2.3.1 VRFCAT

The migration from device resident to cloud-based administration for the VRFCAT did not include alterations of any of the item content, including the graphical depictions and pop-ups identifying stimuli, or instructions, with the same dependent variables collected. Both versions of the VRFCAT were administered in person by an examiner. However, the cloud-based version collected responses from interaction with a touch-screen on an i-Pad.

#### 2.3.2 FUNSAT

Migration from device-resident to cloud-based delivery for the FUNSAT included upgrades in the graphics and a transition to touch screen response entry. Transition from one task to the next was automatically driven by the software based on participant performance and not launched by the examiner. Second, a Spanish-translated version of the software was developed. This translated version included translations of all instructions, feedback, and stimulus materials. The baseline assessments for FUNSAT were completed at an in-person office visit, where the examiner provided only technical support and did not interact with the participant regarding task demands. All training and follow-up assessments with forms B and C were fully remote (Czaja et al., 2024) and were completed on a study provided Chromebook®. Participants were assessed and trained in their commonly spoken language by bilingual testers, either Spanish or English, with all assessments and FUNSAT training performed in the same language.

### 2.4 Procedures

In both programs across both versions, data on all performance parameters, including the primary outcome of completion time, was collected by the program. For the device resident programs, data was manually off-loaded to an encrypted cloud-based database. In the cloud-based versions, performance data was automatically uploaded at the time of assessment.

Participants in the FUNSAT studies were also tested with a computerized brief assessment of cognitive functioning, the BAC-app (Atkins et al., 2017). The BAC-app is a computer-assisted battery that is delivered on a tablet administered by a tester. It is comprised of 6 tests: Verbal Learning, Digit Sequencing (Working memory), Token Motor (motor skills), Verbal fluency (Letter and animal), Symbol Coding (Processing speed), and Tower of London (Executive functioning). Performance on these 6 tests and a composite score were used to examine convergence between completion time on the FUNSAT and cognitive performance.

### 2.5 Data analyses

Analyses compared completion times across the two delivery strategies for the two assessment systems with *t*-tests. The primary outcomes variable, time to completion was the outcome of interest. In addition, descriptive statistics regarding the percentile distributions of the performance scores across administration strategies are presented. We also examined correlations between age and completion times across versions for both VRFCAT and FUNSAT and examined convergence between cognitive performance and completion times in the two FUNSAT studies. A composite cognitive performance score for the BAC-App was developed with Principal Components Analysis, because in the Cloud-based FUNSAT study we needed to combine English and Spanish speakers without access to norms for older Spanish speakers. We also correlated performance on the self-administered remote forms B and C of the FUNSAT with each other and performance on the assessment with form A. These analyses used Pearson correlations.

## 3 Results

### 3.1 Demographics

Table 1 presents the demographic characteristics of the participants pre-and post-migration for each assessment tool. As can be seen in the table, there were very limited differences in demographic characteristics of the participants tested with device-resident and cloud-based versions of the either of the two functional capacity tasks. There was a significant difference in education across the VRFCAT migration studies and there were more individuals of Hispanic ethnicity in the second FUNSAT study because of the efforts to identify and assess Spanish speaking participants.

**Table 1:** Demographic Characteristics of the Two Migration Study Samples of Healthy Controls.

VRFCAT						
	Device-Resident		Cloud-Based			
					X^2^	p
N	348		69			
% Female	54		55		0.026	0.89
% Black Race	20		25		5.08	0.18
					T	p
	M	SD	M	SD		
Age	68.31	8.31	67.36	8.41	0.97	0.33
Education	14.83	2.63	15.68	2.94	2.24	0.028


FUNSAT						
	Device-Resident			Cloud-Based		
					X^2^	p
N	53		72			
% Female	87		92		1.33	0.34
% Black Race	34		29		0.58	0.45
% Hispanic	28		46		6.95	0.008
	M	SD	M	SD	T	p
Age	73.15	5.79	71.17	6.36	1.61	0.11
Education	15.84	2.50	15.56	2.52	0.87	0.39

### 3.2 Completion times: VRFCAT

Table 2 presents the results for completion time on the VRFCAT. The sample assessed with the cloud-based modality had slightly faster (i.e., better) mean time to completion in seconds, but this 5 % difference was not statistically significant: *p* = .13. The variance in completion times did not differ across modality either, when a F test was applied, F = 0.02, *p* = .89. Similarly, there were no differences in completion time when the samples were split by sex or racial status and compared. When a + 5 % linear transformation was applied to the scores on the cloud-based VRFCAT, the scores for performance across percentiles for cloud and vs computer were very similar at the 75th (739 vs 750), 50th (806 vs 830) and 25th (978 vs 1001) percentile cutoffs. A cut-off score corresponding to one SD below the normative mean (17th percentile) was also very similar for cloud vs. computer (1008 vs 1011).

**Table 2:** Task Completion Time for VRFCAT and the Six FUNSAT Tasks Comparing Device-Resident vs. Cloud Based Delivery.

	Device-Resident	Cloud-Based				
**VRFCAT**	*N* = 348	*n* = 69				
**Completion time in Seconds**	M	SD	M	SD	t	p
	840	211	880	200	1.49	0.13
**FUNSAT**	N = 52	*N* = 73				
	M	SD	M	SD	t	p
**Time In seconds**						
Ticket Kiosk	766	324	903	329	2.32	0.02
ATM Banking	906	348	1153	475	3.20	<0.001
Medication	833	311	843	270	0.21	0.83
Management						
Telephone Refill	597	214	695	222	2.48	0.01
Internet Banking	1048	404	1030	395	0.26	0.80
Ugreens Website	1095	486	1183	538	0.94	0.39

### 3.3 Completion times: FUNSAT

Table 2 also presents the results for completion time on the 6 FUNSAT tasks across administration modality. As can be seen in the table, there were significant differences in time to completion on 3 of the 6 tasks, with completion times longer in the cloud-based delivery system. However, participants were assessed in either Spanish or English for the cloud-based system and only English for the device-resident system. When participants assessed in English in the cloud based system (*n* = 48) were compared to the participants with the device-resident system (*n* = 52), the performance differences between delivery systems were small, cloud-based was faster for all subtasks, and the differences were non-significant for the three subtests where performance differed with the full sample: Ticket purchase (*t* = 0.72, *p* = .48), ATM Banking (*t* = 0.77, *p* = .45), and Telephone Voice Menu Refill (*t* = 0.07, *p* = .95).

Using the total completion time across all 6 tasks for English speaking sample and examining distribution standards like the VRFCAT, cut-off scores for device resident vs. cloud-based administration at the 75th (3801 vs 3817), 50th (4744 vs 4794) and 25th (6215 vs 6174) percentiles were very similar. A cut-off score corresponding to one SD below the normative mean (17th percentile) was also very similar for device resident vs. cloud-based (7254 vs 7296).

### 3.4 Correlational analyses

Correlations between age and completion time were essentially identical in the two modalities, *r* = 0.46 and *r* = 0.43, *p* < .001 for the VRFCAT. In line with the VRFCAT analyses, we correlated age and completion time for all 6 simulations and the total score in the two delivery systems. Consistent with the data from VRFCAT migration, there were no differences across delivery systems in terms of correlations with age, although correlations with age and any task, or total completion time, were significant in either sample at a corrected significance level of *p* < .01. The largest correlation was *r* = 0.29, *p* = .02. The somewhat higher mean ages and smaller standard deviations in FUNSAT participants may have reduced the likelihood for age-related correlations.

In contrast to performance on the FUNSAT simulations, there were no significant assessment language differences in performance on any of the 6 BAC-App tests or MOCA scores in the Cloud-Based Study (all *t* < 1.62, all *p* > .07). There were also no MOCA score differences in the samples across the two delivery systems, *t* = 1.06, *p* = .29. We computed Pearson correlations between the BAC-App composite score and FUNSAT performance on the six tasks separately by delivery systems. We included all participants in the cloud-based assessments because there were no differences in NP performance associated with training language.

Table 3 presents these correlations, all of which were statistically significant. None of the correlations differed in their magnitude across delivery systems when compared with r to Z tests. If Spanish speakers were excluded from the correlational analyses, the results were not affected, with all correlations between individual tasks and composite NP performance ranging from *r* = −0.39 to *r* = 0.69, while also not affecting differences in correlations across delivery systems.

**Table 3:** Correlations Between Composite Cognitive Performance Measured with the BAC-App and Completion time for Six FUNSAT Tasks with Device-Resident vs. Cloud Based Delivery.

	Device-Resident		Cloud-Based		
	N = 52	N = 73			
	Pearson r	p	Pearson r	p	*Z*-score for difference
Ticket Kiosk	−0.50	<0.001	−0.40	<0.001	0.67 (*p* = .50)
ATM Banking	−0.68	<0.001	−0.55	<0.001	1.13 (*p* = .25)
Medication	−0.72	<0.001	−0.50	<0.001	1.89 (*p* = .062)
Management					
Telephone Refill	−0.33	0.02	−0.32	0.009	0.06 (*p* = .99)
Internet Banking	−0.57	<0.001	−0.44	<0.001	0.23 (*p* = .82)
Ugreens Website	−0.40	0.004	−0.43	<0.001	0.13 (*p* = .90)
Total Time	−0.63	<0.001	−0.48	<0.001	1.17 (*p* = .24)

Correlations between the different forms of the FUNSAT in the cloud based study are presented in Table 4. Although there is some variation in form to form correlations, largely based on the overall difficulty of the tasks (easier tasks were less strongly correlated across forms), the correlations between forms 2 and 3, administered after training, for individual tasks were largest for all six tests. Cross-form correlations for total completion time across all 6 tasks for the three forms were all over *r* = 0.82. In a non-interventional study in healthy older people (Kim et al., 2024), inter-form correlations for the composite scores of the device resident alternate forms A and B of the VRFCAT were found to be ICC = 0.87, which is very similar to the correlations seen in the cloud-based study.

**Table 4:** Correlations Between Completion time for Six FUNSAT Tasks Across three forms with Cloud Based Delivery.

Fixed Difficulty Form Comparisons			
	B with C	A with B	A with C
Ticket Kiosk	0.81	0.65	0.73
ATM Banking	0.83	0.81	0.76
Medication	0.71	0.62	0.67
Management			
Telephone Refill	0.62	0.59	0.61
Internet Banking	0.82	0.71	0.79
Ugreens Website	0.73	0.65	0.78
Total Time	0.86	0.84	0.82

## 4 Discussion

Two different functional capacity measures were migrated from device resident to cloud-based delivery with slight differences in the migration goals. For both systems, the general finding was that performance was essentially the same on the primary outcomes measure, time to completion, across the two delivery strategies. In terms of the VRFCAT, there was a consistent 5 % advantage for cloud-based delivery that may be due to the transition to entering responses with the touchscreen and not a mouse. FUNSAT performance was essentially the same across delivery strategies when English speakers were examined, with a slight and nonsignificant tendency toward faster completion with cloud-based version with touchscreen entry. Spanish speakers, while not manifesting differences in neuropsychological test performance or MOCA scores, performed more slowly than English speakers on the baseline assessment in the cloud-based delivery system. Since the Spanish version was created during the migration, we do not know if the differences would have been found with the prior version.

For both functional capacity measures, discrimination of the performance of HC and clinical populations, including MCI and SMI, has been previously demonstrated. For the VRFCAT, assessments with the cloud-based for comparison of HC and MCI participants were conducted in a recent study, where MCI participants (tested in English or Spanish on all assessments) performed 0.90 SD more poorly than the HC sample (Harvey et al., 2024). In that study, the average performance difference between MCI participants and the HC participants across all 6 FUNSAT subtests on the cloud-based baseline assessment was very similar to the VRFCAT differences: 0.91 SD. The initial study of FUNSAT performance with device-resident assessments (Czaja et al., 2020) found an average performance disadvantage of 0.83 SD between cognitively impaired and unimpaired participants.

The correlation between cognitive performance and the 6 individual FUNSAT tasks, in the entire HC sample, across languages, ranged from *r* = −0.32 to *r* = −0.55, with the average correlation being *r* = −0.48. These correlations did not differ from those seen with the previous device resident study (Harvey et al., 2021). The correlation between performance on the cloud-based VRFCAT and composite cognition in HC participants was not directly presented in the Harvey et al. (2024) paper, but analysis of the unpublished data revealed a correlation of *r* = −0.40 in the HC sample.

In terms of poorer performance on the part of Spanish speakers on the cloud-based FUNSAT at baseline, analyses of training data from this sample found that training gains on the FUNSAT simulations were larger for Spanish speakers than English Speakers, all d > 0.38 (Macchiarelli et al., 2024). Our interpretation is that our sample of Spanish speakers likely had reduced familiarity with technology-related functional skills prior to study entry, but clearly were able to make considerable training gains with practice. In the Harvey et al. (2024) study of VRFCAT training gains with skills training, there were no baseline differences across languages in VRFCAT performance.

There were several migration updates for both FUNSAT and VRFCAT that were not obvious to participants. These include a third alternative form for FUNSAT and the option to deliver a preselected subset of baseline assessments, rather than all 6 tasks. For both VRFCAT and FUNSAT, downloads of the data were streamlined and made directly accessible to administrators, with the cloud-based databases re-organized to organize downloaded data in an immediately analyzable longitudinal format without data re-organization. Clearly, this would facilitate integration of VRFCAT and FUNSAT data with repeated-measures outcomes data collected in treatment studies.

The limitations of the study include smaller sample sizes for HC in the migrated version of the VRFCAT compared to the prior version and generally smaller samples of HC for the FUNSAT compared to VRFCAT. We did not perform a full mental health diagnostic interview for the participants and cannot rule out influences of depression or anxiety. Error scores are not comparable across FUNSAT and VRFCAT and forced progressions are defined differently in the two systems. The VRFCAT uses a time-based progression and the FUNSAT uses error based progression. Also, the VRFCAT has many fewer actual responses, with some task objectives having only a single response (e.g., “Pick up the billfold”) leading to reduce potential for errors. The FUNSAT has 6 Tasks, with 3–6 subtasks and errors calculated on an item x item within subtasks, leading to a mean number of total errors across 6 simulations for healthy controls in the cloud-based version of 64, compared to 3 for the VRFCAT. No Spanish speaking HC participants were tested in the cloud-based VRFCAT validation study, but there were no baseline language-related differences in VRFCAT performance in the data collected for the subsequent FUNSAT treatment study (Harvey et al., 2024). In person data were used for the baseline scores on the FUNSAT, although convergence with the other forms of the test with remote administration were very high and consistent with the inter-form correlations on the device resident FUNSAT (Kim et al., 2024). An ideal validation study would use a within-subjects design to administer both versions of each task in a counter-balanced order. However, as the device resident software is obsolete, such a study would be challenging to justify.

Migration to cloud-based delivery of functional capacity assessments is clearly feasible, with considerable evidence of similarity across delivery strategies found in the primary outcomes of time to completion. The post-migration VRFCAT is still delivered in person with a human tester, because of its primary intended use as a co-primary measure for FDA-regulated cognitive enhancement studies. Remote delivery of training and reassessment of the FUNSAT has also been shown to be feasible with over 90 % training and follow-up assessment adherence for completely remote FUNSAT delivery (Czaja et al., 2024). These same participants also completed a fully remotely delivered functional assessment using Ecological Momentary Assessment (EMA, Dowell-Esquivel et al., 2024), finding statistically significant everyday functional gains during fully remote training, with increasing gains for the next 3 months.

While it is common to see studies evaluating the migration of paper and pencil cognition and functional capacity assessments to digital delivery, much less information is available about fully digital migrations from device-based to cloud-based delivery. There is considerable information about the Cloud-based FUNSAT in terms of the technical potential for delivery as fully remote, self- administered assessment. Only the baseline assessment was performed in person, suggesting that, for large-scale training programs, the demands on human trainers would be relatively limited compared to the amount of data collected. There are also many participants who have received fully remote access to FUNSAT in on-going clinical intervention programs and have self-administered all elements of assessment and training, although not in systematic clinical trials.

## CRediT authorship contribution statement

SJC, PDH, and PK Designed the FUNSAT studies, obtained funding, and managed the studies.

RSEK Designed the first VRVCAT Studies and obtained funding.

WH designed and supervised the second VRCAT Study.

HK Collected data on the second VRFCAT Study.

PDH Analyzed the data for all studies.

PDH and WH wrote the first draft of the paper.

All authors edited the final version of the paper.

## Funding

Funding was Provided by 10.13039/100000025National Institute of Mental Health Grant Numbers 1R43MH084240 and 2R44MH084240 to Dr. Keefe and National Institute of Aging Grant Numbers 1R21 AG041740 (Czaja and Harvey) and 1 R43 AG057238 and 2 R44 AG057238 (Kallestrup).

## Declaration of competing interest

Dr.’s Harvey and Czaja are co-Chief Scientific Officers of i-Function, Inc. Mr. Kallestrup is Chief Executive Officer of i-Function, Inc.

Dr. Harvey has also received consulting fees or travel reimbursements from Alkermes, Boehringer Ingelheim, Karuna Therapeutics, Merck Pharma, Minerva Neurosciences, and Sunovion (DSP) Pharma in the past year. He receives royalties for BAC-app and BACS (Owned by WCG Endpoint Solutions, Inc. and contained in the MCCB).

Dr. Keefe was CEO at Verasci when these data were collected and now serves as a consultant to WCG, Karuna, Novartis, Kynexis, Gedeon-Richter, Pangea, Merck, and Boehringer-Ingelheim, and receives royalties for the BAC, BACS and VRFCAT.

Dr. Horan was Vice President at Verasci when these data were collected and is now a Full Time Employee of Karuna Therapeutics (A Division of Bristol Myers, Squibb).

Dr. Klein is a full-time employee at WCG Endpoint Solutions (Formerly known as Verasci, Inc).
